# Salivary gland and hand involvement in Systemic sclerosis: A cross-sectional ultrasound and radiographic study

**DOI:** 10.1515/rir-2026-0040

**Published:** 2026-10-22

**Authors:** Theau Cavillon, François Zimmermann, Nicolas Belhomme, Guillaume Coiffier, Pascal Guggenbuhl, Alain Lescoat, François Robin

**Affiliations:** Rheumatology Department, South Hospital, CHU Rennes, University of Rennes, INSERM, Institut NUMECAN (Nutrition Metabolisms and Cancer), Rennes, France; Department of Internal Medicine and Clinical Immunology, CHU Rennes, University of Rennes, INSERM, EHESP, ISRET (Institut de recherche en santé, environnement et travail), Rennes, France; Rheumatology Department, Dinan Hospital Center, Dinan, France

Dear Editor,

Systemic sclerosis (SSc) remains a clinical enigma given its phenotypic heterogeneity observed among affected individuals. SSc-specific antibodies are a valuable tool for patient stratification as some antinuclear antibodies (ANA) profiles (*e.g*. anti-centromere (ACA), anti-topoisomerase I (Scl-70), anti-RNA polymerase III) are associated with distinct clinical phenotypes and specific prognosis. While ANA profiles provide some stratification, it fails to capture the full heterogeneity of disease expression. For example, among patients with anti-Scl-70 antibodies, some develop cutaneous diffuse form while others have a cutaneous limited form, with different clinical outcomes.

A more comprehensive and multimodal phenotyping approach is therefore necessary to better define patient subgroups, prognosis and therapeutic approaches.

Major salivary gland ultrasound (SGUS) is commonly used in the diagnostic evaluation of Sjögren’s disease (SjD), characterized by sicca syndrome (dry eyes, mouth and vagina) which can be also present in SSc patients. The OMERACT (Outcome Measures in Rheumatology) group developed a scoring system for SGUS lesions in patients with SjD.^[1]^ This score ranges from grade 0 (normal parenchyma) to grade 3 (severe change affecting the whole salivary gland). A recent study including 75 SSc patients showed that 48 % of patients with sicca symptoms had a pathological SGUS OMERACT score (grade ≥ 2), while this proportion was 40% in patients without sicca symptoms.^[2]^ However, to date, there are no SGUS signs and related classification or scoring specific to SSc.^[2]^

Hand ultrasound (US) has established itself as a sensitive tool for detecting synovitis or tenosynovitis, including fibrotic tenosynovitis (also called sclerosing tenosynovitis) which is specific to SSc.^[3,4]^ Data from the European Alliance of Associations for Rheumatology (EULAR) Scleroderma Trial and Research Group (EUSTAR) database found that patients with synovitis or sclerosing tenosynovitis displayed a poorer prognosis, regardless of the cutaneous subtype, as demonstrated by the higher prevalence of internal organ complications (interstitial lung disease [ILD], renal crisis, pulmonary arterial hypertension [PAH]) .^[5]^

In this cross-sectional study, integrating SGUS, hand US, and hand radiography, we aimed to find new perspectives on phenotypic subsets in SSc, particularly a potential fibrotic-dominant disease pattern as a unifying mechanism in a subset of patients.

We analyzed a total of 53 SSc patients who underwent SGUS (*n* = 53) and hand US (*n* = 31) and/or hand X-rays (*n* = 51).

Data is presented in Table 1. Notably, 25 (47%) patients exhibited a pathological SGUS OMERACT score (≥2), indicating significant salivary gland involvement, yet no association was found with specific ANA profiles. Hand US revealed synovitis in 5 (16%) patients and tenosynovitis in 6 (19%), with 4 (13%) patients showing sclerosing tenosynovitis. While no association emerged between SGUS and hand US findings, all patients with sclerosing tenosynovitis had a pathological SGUS OMERACT score. While not statistically significant due to the limited number of cases, this observation can suggest a shared fibrotic and/or vascular pathophysiology linking glandular and musculoskeletal structures, independent of autoantibody status. To strengthen this hypothesis, among the 4 patients with sclerosing tenosynovitis and pathological SGUS, skin fibrosis and ILD was more frequent with a median mRSS 10.5 (interquartile range [IQR] 2.25) and 3 (75%) ILD.

**Table 1:** Demographic, clinical and biological characteristics of 53 patients.

Patients’ characteristics (*n* = 53, or specify otherwise)	*N* (%) or Mean (SD) or Median (IQR)
Age (years)	59 (± 14)
Female subject	41 (77)
Height (cm)	165.4 (± 8)
Weight (kg)	65 (IQR = 17)
Smoking status (*n* = 23)	
Never smoker	9 (39)
Current smoker	5 (22)
Fulfilling 2013 EULAR/ACR classification criteria	51 (96)
Overlap syndrom (*n* = 31)	1 (3)
Rheumatoid arthritis	1 (3)
Disease duration (years)(*n* = 52)	8.5 (IQR = 7.25)
Diffuse cutaneous form	11 (21)
Raynaud’s phenomenon duration (years) (*n* = 51)	14 (IQR = 15.5)
History of digital ulcer (*n* = 52)	24 (46)
History of pitting scars	21 (40)
Puffy fingers	45 (85)
Sclerodactyly	30 (57)
Telangiectasia	42 (79)
mRSS (*n* = 52)	3.5 (IQR = 3.25)
Subjective sicca syndrome	36 (68)
ocular	22 (41)
oral (*n* = 48)	33 (63)
Objective sicca syndrome	31 (61)
ocular (*n* = 51)	28 (55)
oral (*n* = 48)	13 (27)
PAH (*n* = 52)	2 (4)
ILD	19 (34)
ILD with DLCO < 70 % (*n* = 52)	12 (23)
History of renal crisis (*n* = 31)	1 (3)
ANA	50 (94)
ACA (*n* = 52)	26 (50)
Scl-70 (*n* = 52)	15 (29)
RNA pol III (*n* = 52)	0
SSA (*n* = 50)	2 (4)
SSB (*n* = 50)	0
OMERACT SGUS score	
0	20 (38)
1	8 (15)
2	10 (19)
3	15 (28)
Pathological (≥2)	25 (47)
Hand US (*n* = 31)	
Synovitis	5 (16)
Tenosynovitis	6 (19)
Sclerosing tenosynovitis	4 (13)

ACR, American College of Rheumatology; ACA, anti-centromere antibodies; ANA, antinuclear antibodies; DLCO, diffusing capacity of the lung for carbon monoxide; EULAR, European League Against Rheumatism; IQR, interquartile range; ILD, interstitial lung disease; mRSS, modified Rodnan Skin Score; OMER-ACT, Outcome Measures in Rheumatology; PAH, pulmonary arterial hypertension; RNA pol III, anti-RNA polymerase III antibodies; Scl-70, anti-topoisomerase antibodies; SD, standard deviation; SGUS, major salivary gland ultrasonography; US, ultrasonography.

Hand X-rays evaluation identified acro-osteolysis in 13 (25%) patients and calcinosis in 10 (20%) patients. These features have been previously associated with disease duration and digital vascular damage,^[6,7]^ aligning with our population which had a median Raynaud’s phenomenon duration of 14 years. However, no clear associations were observed between radiographic abnormalities and SGUS or ANA profiles.

Our study highlights the high prevalence of major salivary gland and musculoskeletal involvement in SSc. These findings generate the hypothesis of distinct SSc subsets that go beyond serological and cutaneous classifications. The observation that some US findings, particularly sclerosing tenosynovitis and pathological SGUS OMERACT score, cluster in the same individuals suggests a fibrotic-dominant disease subset that deserves further investigation. The lack of associations between imaging findings and ANA profiles challenges the traditional serological classification and underscores the importance of integrating imaging data into phenotypic stratification.

Limitations include the monocentric and retrospective design that may have introduced selection bias, limiting generaliz-ability. We selected SSc patients who underwent SGUS and hand imaging, which does not represent the overall SSc population. SjD was not systematically excluded, thus some SGUS abnormalities may reflect SjD rather than SSc pathology. This uncertainty weakens the hypothesis of a specific SSc SGUS-sclerosing tenosynovitis association, which may be related to an overlap syndrom with SjD. The small sample size, particularly for subgroup analyses (*e.g*., patients with sclerosing tenosynovitis or specific ANA profiles), may limit statistical power. However, our study’s integrative design provides a comprehensive view of SSc heterogeneity. Future longitudinal studies with larger cohorts are warranted to validate these observations and explore causal relationships.

In conclusion, our study provides preliminary evidence supporting the concept of distinct SSc subsets defined by shared fibrotic patterns, exceeding serological classifications. Integrating multimodal imaging into phenotypic stratification could lead the way for personalized therapeutic strategies in SSc.

## References

[1] Jousse-Joulin S, D’Agostino MA, Nicolas C (2019). Video clip assessment of a salivary gland ultrasound scoring system in Sjögren’s syndrome using consensual definitions: an OMER-ACT ultrasound working group reliability exercise. Ann Rheum Dis.

[2] Zimmermann F, Robin F, Diot E (2024). Whole phenotype of patients with systemic sclerosis and sicca manifestations: Comparison with sicca manifestations from other causes. Semin Arthritis Rheum.

[3] Elhai M, Guerini H, Bazeli R (2012). Ultrasonographic hand features in systemic sclerosis and correlates with clinical, biologic, and radiographic findings. Arthritis Care Res.

[4] De Saint Riquier M, Ballerie A, Robin F (2020). Hand ultrasound for the diagnosis of scleroderma: a scoring strategy including US items and items from the EULAR/ACR classification. Clin Exp Rheumatol.

[5] Avouac J, Walker U, Tyndall A (2010). Characteristics of joint involvement and relationships with systemic inflammation in systemic sclerosis: results from the EULAR Scleroderma Trial and Research Group (EUSTAR) database. J Rheumatol.

[6] Azarbani N, Javadzadeh A, Mohseni I (2020). Association of Musculoskeletal and Radiological Features with Clinical and Serological Findings in Systemic Sclerosis: A Single-Centre Registry Study. Mediterr J Rheumatol.

[7] Lescoat A, Coiffier G, de Carlan M (2018). Combination of Capillaroscopic and Ultrasonographic Evaluations in Systemic Sclerosis: Results of a Cross-Sectional Study. Arthritis Care Res.

[8] Leymarie S, Bahans C, Paltoglou M (2022). Ethical and legal considerations in non interventional health clinical trials in the French context. Contemp Clin Trials Commun.

